# Reversion of epithelial-mesenchymal transition by a novel agent DZ-50 via IGF binding protein-3 in prostate cancer cells

**DOI:** 10.18632/oncotarget.19659

**Published:** 2017-07-28

**Authors:** Zheng Cao, Shahriar Koochekpour, Stephen E. Strup, Natasha Kyprianou

**Affiliations:** ^1^ Department of Urology, University of Kentucky, Lexington, KY, USA; ^2^ Department of Genetics and Genomic and Urology, Roswell Park Cancer Institute, Buffalo, NY, USA; ^3^ Departments of Biochemistry and Toxicology & Cancer Biology, University of Kentucky, Lexington, KY, USA

**Keywords:** prostate stroma, targeted therapeutics, mesenchymal changes, tumor microenvironment, DZ-50

## Abstract

Dysregulation of transforming growth factor-β1 (TGF-β1) and insulin-like growth factor (IGF) axis has been linked to reactive stroma dynamics in prostate cancer progression. IGF binding protein-3 (IGFBP3) induction is initiated by stroma remodeling and could represent a potential therapeutic target for prostate cancer. In previous studies a lead quinazoline-based Doxazosin® derivative, DZ-50, impaired prostate tumor growth by targeting proteins involved in focal adhesion, anoikis resistance and epithelial-mesenchymal-transition (EMT). This study demonstrates that DZ-50 increased expression of the epithelial marker E-cadherin, and decreased the mesenchymal marker N-cadherin in human prostate cancer cells. In DU-145 cells, the effect of DZ-50 on EMT towards mesenchymal epithelial transition (MET) was inhibited by talin1 overexpression, a focal adhesion regulator promoting anoikis resistance and tumor invasion. DZ-50 treatment of human prostate cancer cells and cancer-associated fibroblasts (CAFs) downregulated IGFBP3 expression at mRNA and protein level. In TGF-β1 responsive LNCaPTβRII, TGF-β1 reversed DZ-50-induced MET by antagonizing the drug-induced decrease of nuclear IGFBP3. Furthermore, co-culture with CAFs promoted prostate cancer epithelial cell invasion, an effect that was significantly inhibited by DZ-50. Our findings demonstrate that the lead compound, DZ-50, inhibited the invasive properties of prostate cancer epithelial cells by targeting IGFBP3 and mediating EMT conversion to MET. This study integrated the mechanisms underlying the effect of DZ-50 and further supported the therapeutic value of this compound in the treatment of advanced metastatic prostate cancer.

## INTRODUCTION

Prostate cancer accounts for 21% of cancer diagnoses and 8% of cancer-related deaths in men with a total of 180,890 new cases and 26,120 deaths estimated in 2016 in the United States [1]. Advances in diagnostic, surgical and radiotherapy approaches have been shown to have a significant impact on impairing tumor progression and improve patient survival [2]. Androgen deprivation therapy (ADT) including medical or surgical castration remains the primary treatment option for prostate cancer patients with metastatic disease [3, 4]. However, after ADT the majority of patients eventually develop castration-resistant prostate cancer (CRPC) [4, 5]. The recognition that CRPC is driven by aberrant androgen signaling and androgen receptor (AR), led to the utilization of AR-directed second generation antiandrogens, abiraterone and enzalutamide for the treatment of metastatic CRPC (mCRPC) [5, 6]. Taxanes, including docetaxel and cabazitaxel, are 1^st^ and 2^nd^ line chemotherapy respectively, for the treatment of patients with mCRPC developing resistance to ADT [7], and contribute to only a modest improvement of survival [8–10]. Overriding this resistance requires understanding of the driving mechanisms, besides the AR in CRPC in the context of the tumor microenvironment and development of targeted therapeutics.

The heterogeneity characterizing the prostate gland consists of luminal epithelial cells, basal cells and a small number of neuroendocrine cells among the epithelium in the surrounding stroma [11]. The reactive prostate stroma within the microenvironment functionally contributes to prostate cancer initiation, progression and metastasis [12]; with stroma-derived transcriptome signatures shown to be of strong predictive value of patient survival, disease progression and therapeutic resistance [12]. Normal fibroblasts maintain tissue homeostasis, however, myofibroblasts and cancer-associated fibroblasts (CAFs) facilitate tumor progression via their repair-centric system and pro-survival biology, promoting tumor growth and vascularity [13]. Taking control of the dynamics of prostate stroma microenvironment is the multifunctional cytokine transforming growth factor-β1 (TGF-β1); activation of TGF-β1 signaling mediates the transition of fibroblast to myofibroblasts [14] and promotes prostate cancer cell-mediated differentiation of normal fibroblasts into CAFs towards metastasis [13, 15].

Additional growth factor signaling networks contextual to the tumor microenvironment are functionally involved in prostate tumor growth and progression including the insulin growth-factor (IGF) signaling axis [16, 17]. IGF ligand binds to the IGF-I receptor, a transmembrane tyrosine kinase receptor, and subsequently IGF-II receptor to activate downstream signaling cascades, such as AKT, promoting cell proliferation and survival [16]. Stimulation of the IGF-I axis up-regulates zinc finger E-box-binding protein1 (ZEB1) which promotes the process of epithelial-mesenchymal-transition (EMT) in human prostate cancer cells [18]. EMT is characterized by loss of epithelial protein E-cadherin and upregulation of mesenchymal proteins (N-cadherin and vimentin); ZEB1 facilitates EMT by acting as a transcriptional repressor of E-cadherin expression [18], towards invasion and metastasis. A clinically significant increase in serum and prostatic tissue levels of IGF ligands has been detected in prostate cancer patients [16]. IGFs also bind to IGF-binding proteins (IGFBPs), with IGFBP3 being the most abundant carrier of IGF ligands in serum [16, 17]. IGFBP3 inhibits IGF signaling due to high-affinity binding to IGFs and subsequent sequestration of the ligand or due to IGF-independent mechanisms [16, 17]. Moreover, IGFBP3 can promote IGF action in diverse human cells including skin fibroblasts [19], breast carcinoma cells [20] and prostate cancer cells overexpressing a constitutively active AR [21]. Mechanistic dissection in cultured fibroblasts indicated that IGFBP3 potentiates IGF action via the phosphatidylinositol-3-kinase pathway [22]. In human esophageal epithelial cells, IGFBP3, in an IGF independent mechanism, promotes TGF-β1-mediated EMT and activates transcriptional regulators essential in EMT, including snail, ZEB1 and ZEB2 [23]. Specifically, IGFBP3 loss suppressed TGF-β1-mediated EMT, while IGFBP3^I56G/L80G/L81G^, a mutant IGFBP3 lacking IGF binding capacity, prevented the IGFBP3 knockdown effect. Furthermore, IGFBP3^I56G/L80G/L81G^ promoted EMT *in vivo* in a Ras-transformed esophageal xenograft model [23], implicating an IGF independent action of IGFBP3 to promote EMT.

Our previous studies on the structural optimization of the quinazoline-based α1-adrenoceptor antagonist Doxazosin®, led to the generation of a lead derivative, DZ-50, that impaired prostate tumor growth through anoikis [24, 25]. A genome-wide microarray analysis and pathway association analysis of pre-clinical models [24] identified that DZ-50 down-regulated genes encoding regulators of extracellular matrix (ECM), tight junctions, angiogenesis, and a component of IGF axis involved in prostate stroma remodeling (IGFBP3) [14]. The present study focused on characterizing the role of IGFBP3 in TGF-β1-mediated EMT and reversal to MET in response to the drug. We found that DZ-50 antagonized TGF-β1-promoted cell invasion by targeting IGFBP3 in both prostate cancer epithelial cells and CAFs, supporting its therapeutic value.

## RESULTS

### Induction of MET in prostate cancer epithelial cells by DZ-50

**Figure 1:**
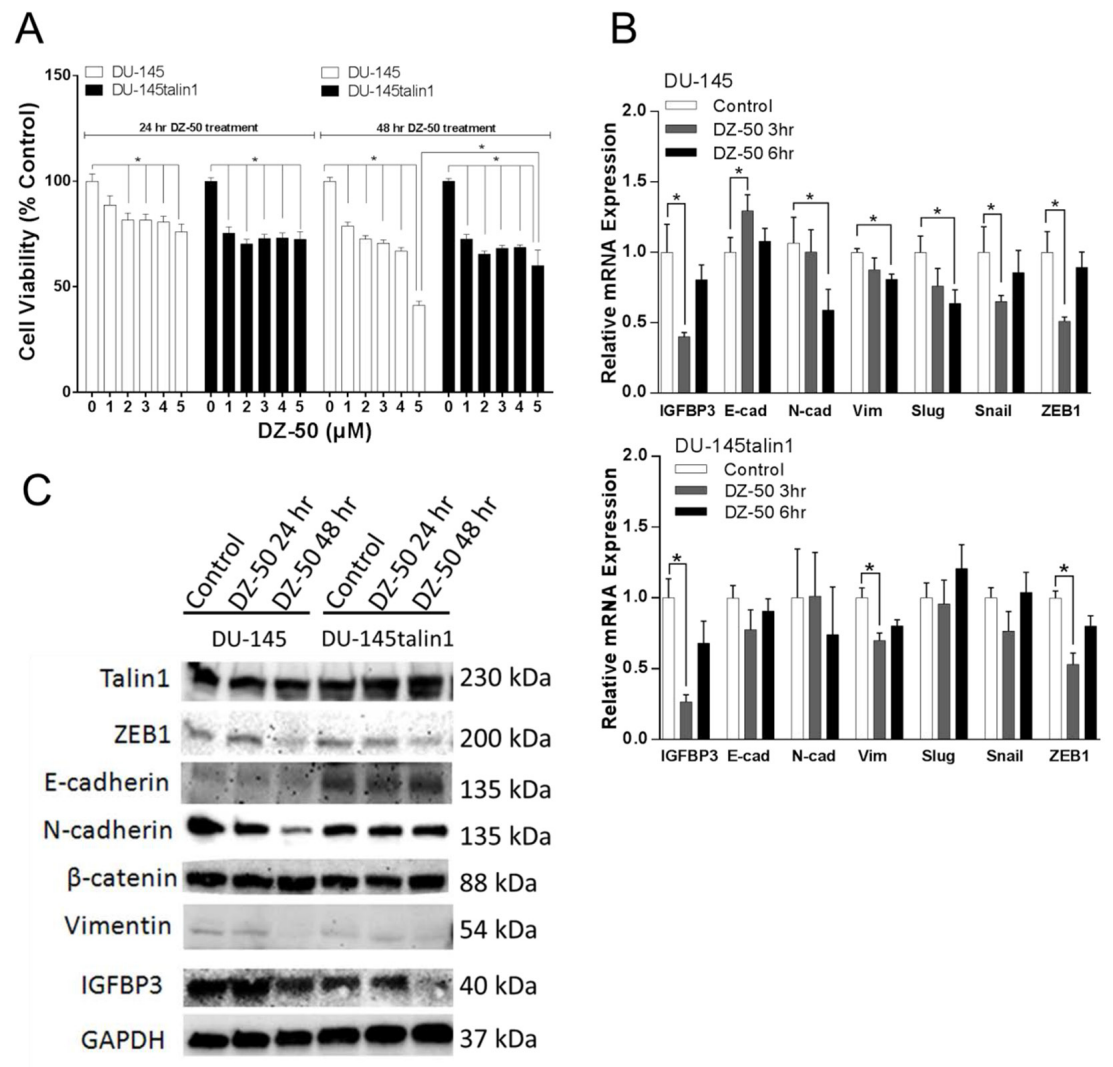
Effect of DZ-50 on prostate cancer cell death and EMT **Panel A**, indicates the response of human prostate cancer cells to increasing concentrations of DZ-50. Cell viability was inhibited in both DU-145 and DU-145talin1 cells in a dose- and time-dependent manner by DZ-50, n=4;*p<0.05. **Panel B**, reveals the mRNA expression profile of EMT regulators IGFBP3, E-cadherin (E-cad), N-cadherin (N-cad), vimentin (Vim), Slug, Snail and ZEB1 in response to DZ-50 (2µM). mRNA levels were determined by RT-PCR; n≥6; *p<0.05. **Panel C**, Effect of DZ-50 on IGFBP3, E-cadherin, N-cadherin, β-catenin, vimentin, talin1 and ZEB1 protein expression (by Western blot).

Cell viability of DU-145 and DU-145talin1 was decreased by DZ-50 in a concentration dependent manner (Figure 1A). Our previous studies demonstrated that DZ-50 at 5µM downregulated talin1 expression and drug-induced anoikis was prevented by talin1 overexpression [24]. In the current study, the ability of DZ-50 to reduce cell viability after 48hrs of treatment was compromised by talin1 overexpression. The effect of DZ-50 on mRNA expression of IGFBP3, E-cadherin, N-cadherin, Vimentin, Slug/Snail (E-cadherin repressor), and ZEB1 was subsequently profiled (Figure 1B). Within 3hrs of treatment there was a significant decrease in IGFBP3 mRNA expression, and an increase in E-cadherin mRNA. A temporal decrease in mRNA levels for N-cadherin, vimentin and slug was detected after 6hrs of exposure to the drug. DZ-50-induction of E-cadherin mRNA and downregulation of N-cadherin, slug and snail mRNA were prevented by elevated talin1 (Figure 1B). Phenotypic EMT profiling in DU-145 and DU-145talin1 cells revealed that DZ-50 (2µM; 48hrs) decreased IGFBP3, N-cadherin, ZEB1 and vimentin protein expression (Figure 1C). The effect of DZ-50 on N-cadherin and vimentin was antagonized by talin1 overexpression (Figure 1C).

**Figure 2:**
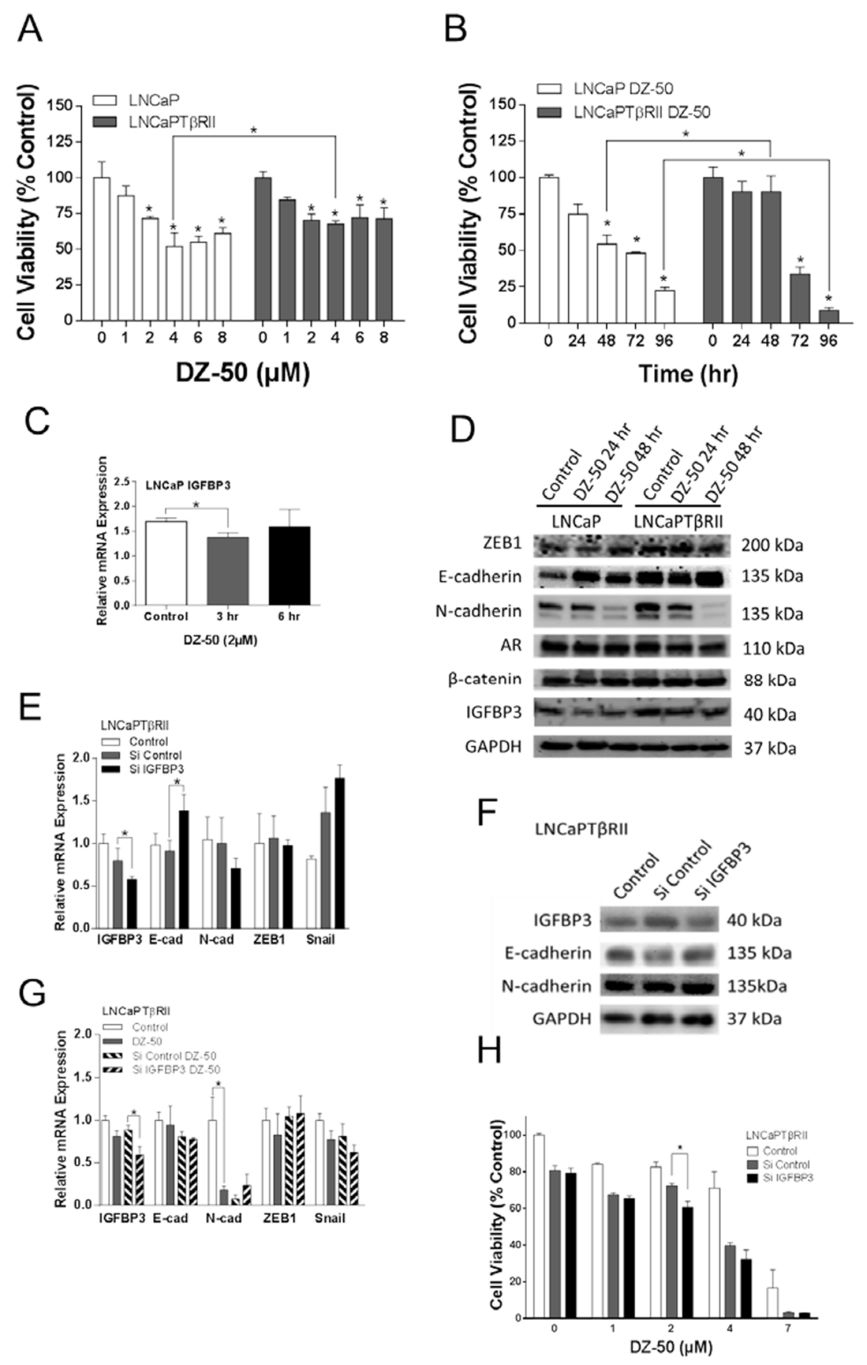
Functional involvement of IGFBP3 in the reversion of EMT to MET in prostate cancer cells **Panels A and B**, reveal the dose response and time course of DZ-50 treatment of prostate cancer cells LNCaP and LNCaPTβRII. DZ-50 decreased cell viability in both cell lines; n=4; *p<0.05 compared to control. **Panel C**, indicates the effect of DZ-50 (4 µM) on IGFBP3 mRNA; n = 6. *p<0.05. **Panel D**, Western blot analysis of ZEB1, E-cadherin, N-cadherin, AR, β-catenin, and IGFBP3 protein levels in response to DZ-50 (4 µM, 24-48hrs). **Panels E and F**, shows the consequences of IGFBP3 loss on EMT regulators mRNA (by RT-PCR) and protein expression (by Western blot); n = 5. *p<0.05. **Panel G**, the effect of DZ-50 (3hrs; 4µM) on mRNA expression after IGFBP3 knockdown (n=5; *p<0.05). **Panel H**, reveals the effect of IGFBP3 silencing on LNCaPTβRII cell response to DZ-50 (4µM, 48hrs); n=3; *p<0.05.

The human prostate cancer cells LNCaP, lack TGF-β type II receptor and thus are refractory to effect of TGF-β1; genetically-engineered overexpression of the type II receptor restores sensitivity of LNCaP cells to TGF-β1 [26, 27]. Treatment with DZ-50 significantly inhibited cell viability in both the LNCaP and LNCaPTβRII cells in a concentration- and time-dependent manner (Figure 2, panels A and B respectively); this effect of the drug was antagonized by TGF-β signaling in the LNCaPTβRII cells after 48 hrs of exposure to DZ-50. Further reduction in cell viability in these cells was observed after 96hrs of treatment. Expression profiling of the candidate targets revealed that DZ-50 decreased IGFBP3 mRNA expression within 3hrs of treatment (Figure 2C) with a consequential decrease in IGFBP3 protein levels (by 24hrs) (Figure 2D) in the LNCaP, but not in the LNCaPTβRII cells. There was reduced N-cadherin protein expression, paralleled by elevated E-cadherin after 48hrs of drug exposure (Figure 2D), indicating a reversal to epithelial phenotype (MET). The intrinsically active TGF-β signaling in the LNCaPTβRII prostate cancer cells antagonized the inhibitory effect of DZ-50 on IGFBP3 expression. Expression of ZEB1, AR and β-catenin was not affected by the drug (Figure 2D).

We subsequently investigated the functional consequences of IGFBP3 loss on the sensitivity of LNCaPTβRII cells to DZ-50. As shown on Figure 2 (panels E and F), IGFBP3 knockdown led to E-cadherin mRNA and protein upregulation and a modest N-cadherin mRNA decrease. The effect of IGFBP3 loss on E-cadherin mRNA was prevented by DZ-50 exposure (Figure 2G). No significant differences were detected in mRNA expression for Snail and ZEB1 by IGFBP3 knockdown regardless of the presence of DZ-50 (Figure 2E and 2G). IGFBP3 knockdown however enhanced the sensitivity of LNCaPTβRII cells to the drug, further reducing cell viability (Figure 2H).

### DZ-50 decreased nuclear expression of IGFBP3 in LNCaPTβRII cells

**Figure 3:**
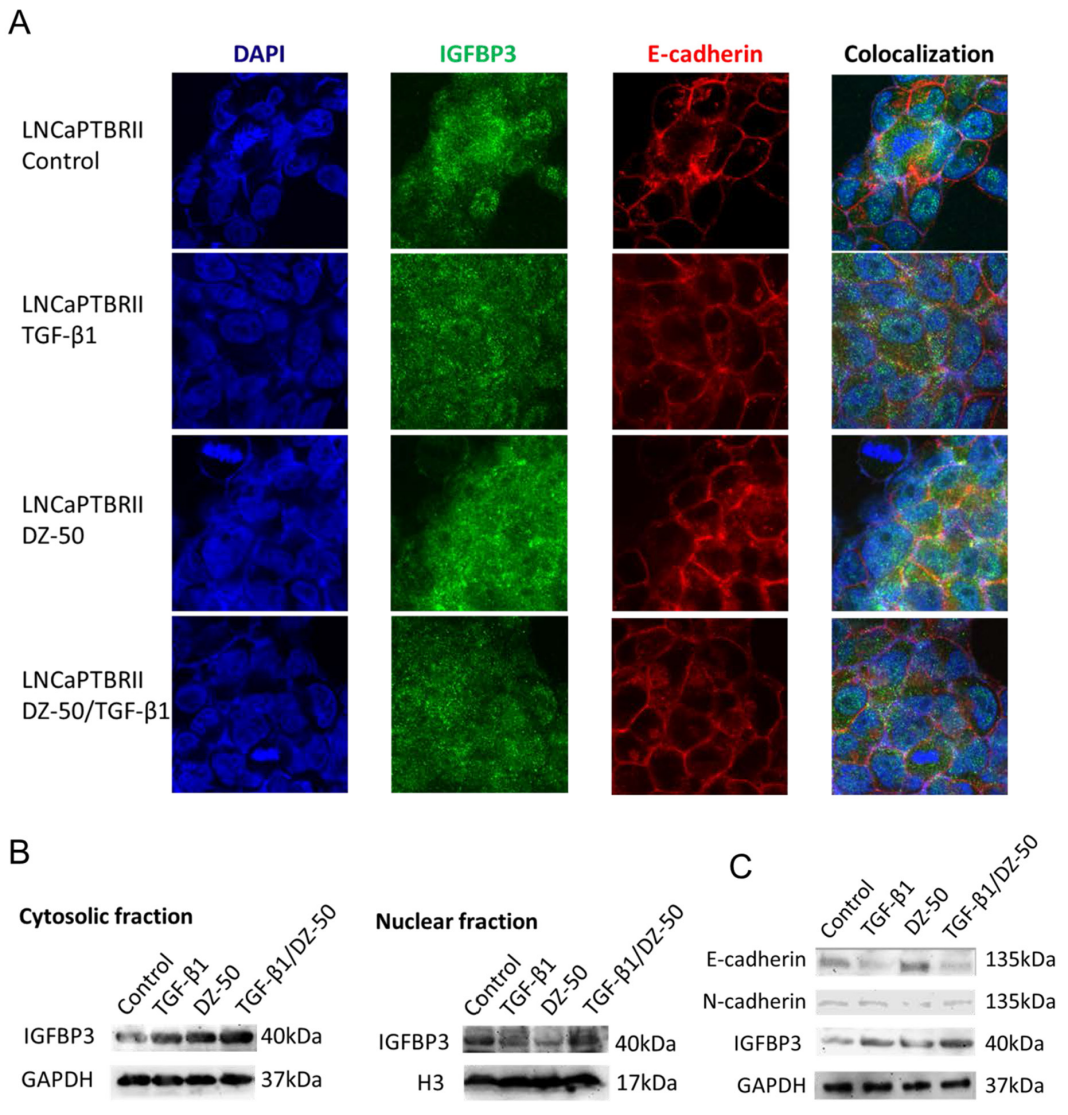
Effect of DZ-50 on nuclear IGFBP3 in LNCaPTβRII cells **Panel A**, representative confocal images of IGFBP3 and E-cadherin localization in LNCaPTβRII cells. Cells were exposed to DZ-50 (24hrs), in the absence or presence of TGF-β1 (5ng/ml) and subjected to fluorescent labeling for IGFBP3, E-cadherin and DAPI. Magnification, X60. **Panel B**, Western blot analysis of subcellular fractions for IGFBP3 protein after treatment with DZ-50 (48hrs) in presence/absence of TGF-β1 (5ng/ml). **Panel C**, Western blot of E-cadherin, N-cadherin and IGFBP3 levels after DZ-50 exposure (4µM, 48hrs) in the presence/absence of TGF-β1. The blot is representative of three independent experiments.

LNCaP and LNCaPTβRII cells were treated with DZ-50 (4µM) for 24hrs in the presence or absence of TGF-β1 (5ng/ml), and subjected to confocal microscopy. For both the LNCaP and LNCaPTβRII cells, E-cadherin was primarily localized on cell membrane regardless of the presence of the drug (Supplementary Figure 1A and Figure 3A). IGFBP3 was distributed in both cytoplasmic and nuclear fractions of the cells (Supplementary Figure 1A and Figure 3A), with a predominant nuclear localization in LNCaPTβRII cells (Figure 3A). DZ-50-induced membrane localization of IGFBP3 and E-cadherin was inhibited by TGF-β1 in these cells (Figure 3A); in LNCaP cells there was no apparent effect by TGF-β1 (Supplementary Figure 1A).

Western blot analysis of protein in subcellular fractions from LNCaPTβRII cells, revealed that TGF-β1 increased cytosolic IGFBP3 levels with no effect on nuclear protein. Treatment with DZ-50 decreased IGFBP3 expression in the nuclear, while it increased IGFBP3 levels in the cytosol (Figure 3B). DZ-50 treatment in the presence of TGF-β1, sustained elevated cytosolic IGFBP3, but had no effect on nuclear levels. The above change of the fractional expression of IGBFP3 resulted in the diffused distribution of the protein by the drug alone or in combination with TGF-β1 as shown by confocal microscopy (Figure 3A). In LNCaP cells, DZ-50 decreased nuclear IGFBP3 levels, with no apparent effect on cytosolic protein levels regardless of TGF-β1 presence (Supplementary Figure 1B). There was no effect on IGFBP3, AR, E- and N-cadherin in LNCaP cells by TGF-β1 (Supplementary Figure 2). TGF-β1 led to reduced E-cadherin and increased total IGFBP3 expression in LNCaPTβRII cells, while it reversed DZ-50-induced N-cadherin decrease (Figure 3C). Smad4 expression was not affected by DZ-50 (Supplementary Figure 3).

### DZ-50 decreased IGFBP3 expression in CAFs

**Figure 4:**
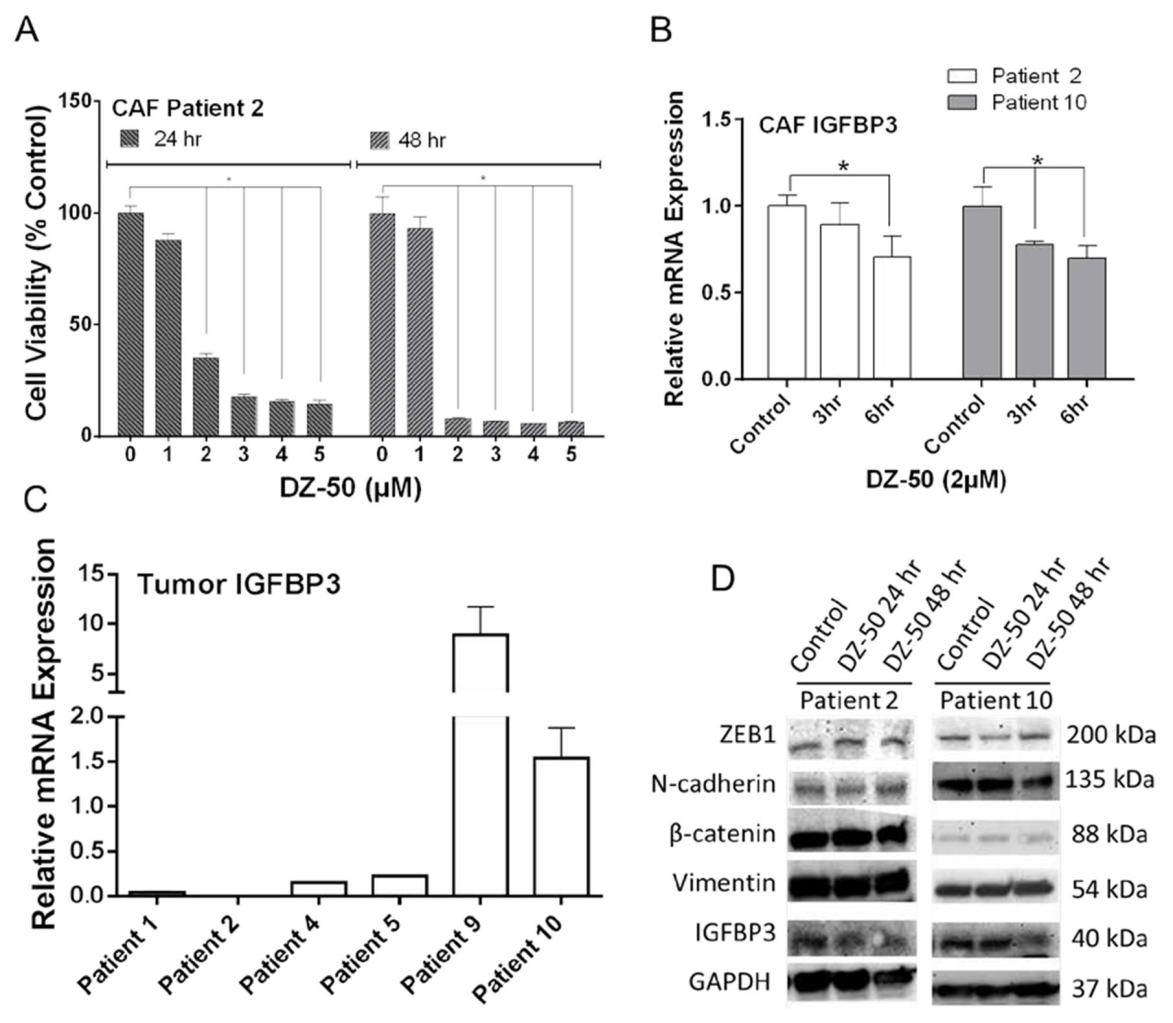
CAFs Response to DZ-50 **Panel A**, shows the effect of DZ-50 on cell viability in CAFs derived from Patient 2. DZ-50 decreased cell viability in CAFs in a dose- and time-dependent manner, n=4; *p<0.05. **Panel B**, expression profile of IGFBP3 mRNA in CAFs derived from Patient 2 and 10 in response to DZ-50, n≥6; *p<0.05. **Panel C**, IGFBP3 mRNA expression in prostate tumors. **Panel D**, shows the expression profile for IGFBP3, N-cadherin, β-catenin, vimentin and ZEB1 proteins in patient-derived CAFs after DZ-50 treatment (2µM, 24-48hrs).

Cell viability of CAFs derived from Patient 2 and 9 (Supplementary Table 1) was decreased by DZ-50 in a dose- dependent manner (Figure 4A; Supplementary Figure 4C). CAFs derived from Patient 9 were more resistant to DZ-50 treatment compared to those from Patient 2 (Figure 4A; Supplementary Figure 4C). DZ-50 (2µM) inhibited IGFBP3 mRNA expression in CAFs derived from Patient 2 and Patient 10 (Supplementary Table 1) after 6hrs, and 3/6hrs treatment, respectively (Figure 4B). The drug had no effect on IGFBP3 mRNA/protein expression in CAFs derived from Patient 9 (Supplementary Figure 4A and 4B). The highest IGFBP3 mRNA level was detected in prostate tumor with the highest Gleason score (4+5) (Figure 4C). DZ-50 treatment decreased IGFBP3 protein expression in CAFs (derived from Patient 2 and 10), but had no significant effect on ZEB1, N-cadherin, β-catenin, or vimentin levels (Figure 4D).

### DZ-50 inhibited DU-145 and LNCaP cell migration in CAF co-culture

**Figure 5:**
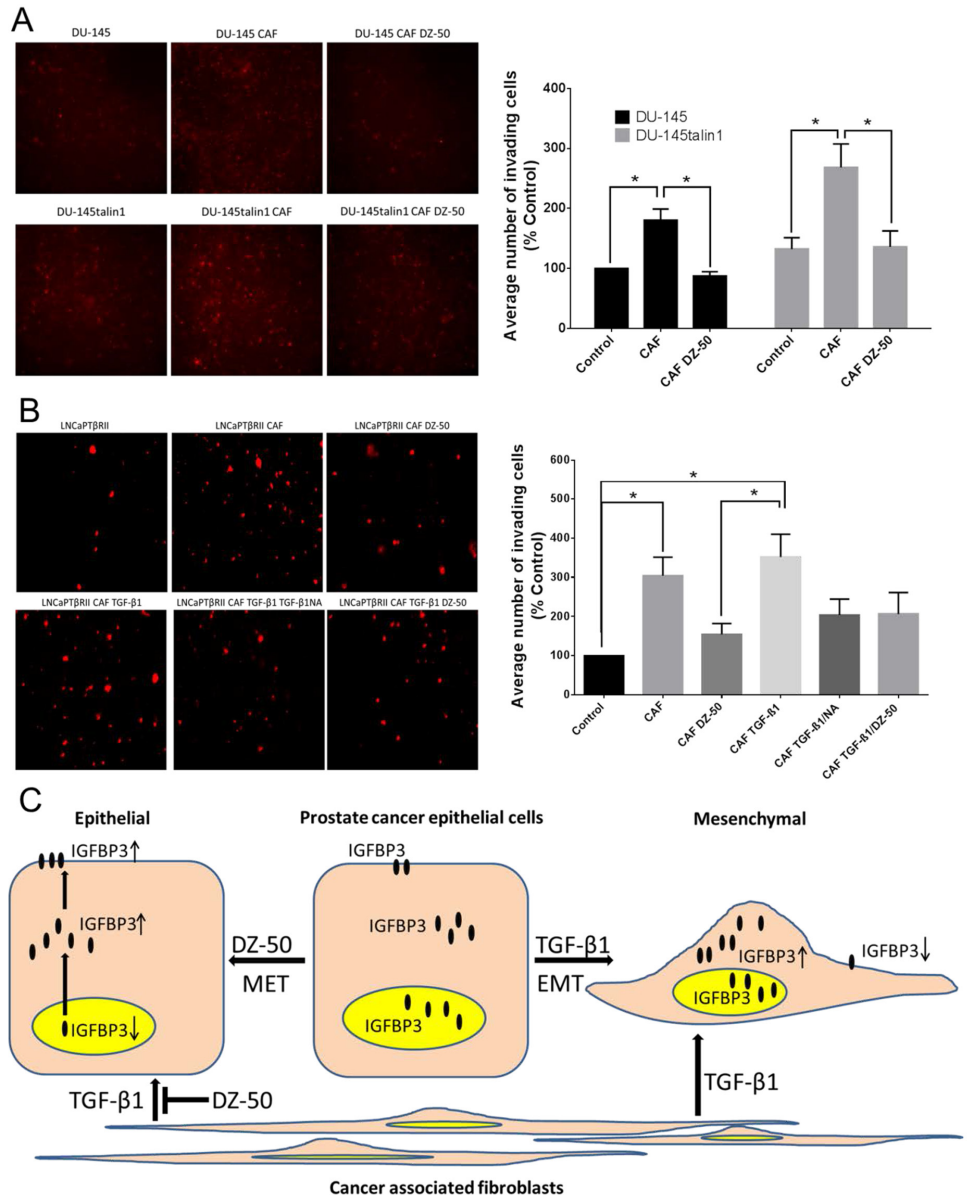
Effect of DZ-50 on CAF-mediated migration of prostate cancer cells **Panel A**, shows fluorescent images of migrated DU-145 and DU-145talin1 cells co-cultured with CAFs in the absence/presence of DZ-50 (2µM; 24hrs) and respective numerical data from quantitative analysis. **Panel B**, shows fluorescent images of migrated LNCaPTβRII cells co-cultured with CAFs in the absence/presence of the drug (2µM; 24hrs), TGF-β1 (5ng/ml; 24 hrs), and TGF-β1NA (TGF-β1 neutralizing antibody; 500ng/ml); numerical data were analyzed as described in “Materials and Methods”; Magnification x40; *p<0.05. **Panel C**, schematic illustration of pathway via which DZ-50 dictates EMT to MET conversion and CAF-facilitated prostate cancer cell migration via targeting nuclear localization of IGFBP3.

In order to recapture the stroma component of the prostate tumor microenvironment, co-cultures of CAFs and prostate cancer epithelial cells were used to evaluate the effect of DZ-50 on cell migration. As shown on Figure 5 (panel A), co-culture of DU-145 and DU-145talin1 cells with prostate CAFs promoted prostate cancer cell migration, an effect that was impaired by DZ-50 (2µM). The CAF-mediated-cell migration in the androgen-sensitive cell lines LNCaPTβRII (Figure 5B), and LNCaP (Supplementary Figure 5), was significantly inhibited by DZ-50 treatment. In the LNCaPTβRII cells, the ability of TGF-β1 to directly promote prostate cancer cell migration was also blocked by DZ-50 (Figure 5B).

## DISCUSSION

This study provides the first evidence on the impact of our lead quinazoline agent, DZ-50, on EMT reversion to the MET phenotype in different human prostate cancer cell lines and in co-culture cell models (with CAFs), via targeting IGFBP3. The drug-induced MET was prevented by talin1 overexpression in DU-145 androgen-independent prostate cancer cells, in accord with recent evidence suggesting that talin1 loss impaired EMT and consequential acquisition of cell motility [28]. DU-145 talin1 overexpressing cells developed resistance to DZ-50-induced anoikis [24] and acquisition of the mesenchymal phenotype induced by talin1 overexpression was associated with the anoikis resistance [29]. In addition, phosphoinositide-generating enzyme, PIPKIγ, and talin1 together control the adhesion and phosphoinositide signaling that regulates EMT [28], indicating that important coupling enzymes in addition to talins are required for EMT induction. The involvement of talin1 in cadherin regulated cell-cell attachment is supported by evidence suggesting that a novel calpain-dependent proteolytic cleavage of talin1 results in the release of a 70-kD C-terminal fragment that rescues the cell-cell adhesion formation [30]. One could thus easily argue that talin1 overexpression might generate more calpain-dependent proteolytic cleaved protein, recruiting E-cadherin to the cell membrane. Interestingly enough, in DU-145 cells, E-cadherin was not upregulated by DZ-50, which could be due to posttranslational modification such as protein degradation. In that context one may consider that several E3 ubiquitin ligases are involved in EMT regulation, and genetic alterations of these ligases have been found in prostate cancer [31, 32].

TGF-β1 promotes prostate tumor migration, invasion and metastatic spread through navigating functional interactions within the tumor microenvironment [10]. Co-culture with CAFs promoted prostate cancer epithelial cell migration and invasion via TGF-β1, consistent with a TGF-β1-induced EMT phenotype, which was pharmacologically inhibited (by DZ-50). In the TGF-β1-responsive LNCaPTβRII cells, DZ-50 promoted MET by downregulating nuclear IGFBP3, an action antagonized by TGF-β1. Considering that IGFBP3 promotes TGF-β1-mediated EMT by activating critical transcriptional regulators, including Snail and ZEB1 in human esophageal epithelial cells [23], the DZ-50 targeting of nuclear IGFBP3 could be driving the drug-induced MET. The present findings provide new insights into the ability of TGF-β1 to modify the response of prostate cancer cells to DZ-50. Mechanistically, TGF-β1 previously shown to upregulate IGFBP3 [23, 26], may directly antagonize the effect of DZ-50 on IGFBP3 and prevent pharmacologically-induced MET (schematically shown on Figure 5C). Functional silencing of IGFBP3 in prostate cancer epithelial cells resulted in increased E-cadherin, while neither of the transcriptional repressors, Snail or ZEB1 mRNA expression was significantly affected (by IGFBP3 knockdown) begging the question as to its role in EMT-MET cycling. An alternative pathway through which IGFBP3 regulates EMT, might involve interaction of nuclear IGFBP3 with the E-cadherin DNA promoter. Such a possibility gains support from reports in other systems indicating the interaction of IGFBP3 with nuclear receptors (retinoid receptors, PPARγ and Nur77), as well as the histone-DNA complex [33]. Import of IGFBP3 into the nucleus is mediated by binding to importin-β (i.e., Karyopherin-β) to form the importin-α/β nuclear transport complex, sharing a common pathway with IGFBP5 [34–36]. The focus on nuclear localization of IGFBP3 takes particular significance as the nuclear protein (IGFBP3) has been previously associated with human prostate cancer recurrence [37]. TGF-β1 prevented the effect of DZ-50 on nuclear IGFBP3 and EMT reversal, supporting the involvement of nuclear IGFBP3 in promoting EMT [10], in accord with evidence that IGFBP3 enhanced TGF-β1-mediated fibroblast differentiation towards EMT [14]. Here we propose a new effect of DZ-50 on nuclear IGFBP3 mediating the pharmacologic reversion of EMT to MET in prostate cancer cells (Figure 5C). The impact of DZ-50 treatment on this nuclear transport dynamic is currently being investigated in pre-clinical models of prostate cancer progression.

In esophageal squamous cell carcinoma, IGFBP3 promotes tumor progression in a subset of tumor cells with a concurrent high expression of CD44 [38]. CD44, a major cell surface hyaluronic acid receptor, is involved in invasion, metastasis and drug resistance in many human malignancies including prostate cancer [38, 39]. High CD44 expression is a characteristic of cancer stem cells functionally linked to prostate cancer metastasis [40]. Switch from CD44+ cell to EMT cell is regulated by TGF-β1-CD44 signaling as a critical step in prostate cancer cell metastasis [41]. Thus one can speculate on a potential engagement of IGFBP3 by the TGF-β1-CD44 signaling of EMT in prostate tumors. In addition, the functional contribution of IGFBP3 to prostate cancer cell apoptosis [42], involves the IGFBP3-mediated translocation of Nur77 from the nucleus to mitochondria [43]. Moreover while IGFBP3 enhanced doxorubicin-induced apoptosis, it also promoted survival under serum starvation, indicating a dual role for IGFBP3 in human endothelial cells [44]. At the molecular level, IGFBP3 activated SphK1 and led to S1P-dependent transactivation of EGFR in normal mammary epithelial and breast cancer cells [45, 46]. SphK1 activation by IGFBP3 has been implicated in resistance to chemotherapy-induced DNA damage by forming nuclear complexes with EGFR and DNA-PKcs [47]. The IGF axis is involved in prostate cell proliferation, differentiation and apoptosis [48, 49]. Clinical evidence has established a positive correlation between elevated IGF-1Rs in prostate stroma tissue and Gleason grade, and down-regulation of epithelial IGFBP3 in prostate cancer patients [50]. Positioning a role for IGFBP3 in the tumor microenvironment, elevated IGFBP3 in stroma tissue was found in pre-clinical models of prostate cancer resonating with the evidence in human prostate cancer specimens [23, 51].

Prostate stroma heterogeneity in prostate cancer could account for the differential response of CAFs (from different patients) to DZ-50. CAFs derived from high grade prostate tumor harboring the highest IGFBP3 mRNA levels, exhibited resistance to the drug, compared to CAFs from low-grade tumors. The differences in the response of CAFs from patient 2 vs patient 9 to DZ-50, as well as the differential IGFBP3 mRNA expression among the six prostate tumors (from individual patients), could be driven by the stroma and tumor heterogeneity that characterizes prostate tumors and the microenvironment [52, 53]. Thus heterogeneity in tumor stroma constitution and its functional interaction with tumor epithelial cells may “calibrate” therapeutic sensitivity. A better understanding of functional interactions between fibroblasts and tumor epithelial cells, as mediated by IGFBP3, will define the role of stroma to therapeutic resistance in advanced prostate cancer [53].

Our findings taken together with clinical evidence that IGFBP3 promoter polymorphism contributes to prostate cancer risk [54, 55], and its potential biomarker value in prostate cancer patients [56], support the clinical significance of IGFBP3. This study enhances our understanding of a new dynamic in the prostate tumor stroma involving IGFBP3 as an EMT regulator, and its impact on the therapeutic response of cancer epithelial cells. The effect of the new agent, DZ-50, on MET induction and TGF-β1-mediated prostate tumor cell migration by targeting IGFBP3, may facilitate a new therapeutic optimization platform for the treatment of advanced CRPC.

## MATERIALS AND METHODS

### Cell cultures and drugs

Human prostate cancer cell lines PC-3, DU-145, LNCaP were obtained from the American Type Tissue Culture Collection (Rockville, MD). Primary prostatic CAFs are provided by Dr. S. Koochekpour (Roswell Park Cancer Institute, Buffalo, NY). The LNCaP cells overexpressing the TGF-β receptor II (LNCaPTβRII) [26], DU-145 cells overexpressing talin1 (DU-145 talin1) [57], and prostate cancer cells co-cultured with primary prostatic CAFs [58] have been established in our laboratory. DZ-50, a first-generation doxazosin quinazoline derivative was used as the novel therapeutic agent [25].

### Antibodies

Antibodies against specific proteins were obtained as follows: The monoclonal antibody against N-cadherin from Abcam (San Francisco, CA); for E-cadherin from BD Biosciences (San Jose, CA); antibodies against the human β-catenin and vimentin from Cell Signaling Technology (Beverly, MA); monoclonal antibodies against ZEB1 and IGFBP3 were from the Bethyl Laboratories (Montgomery, TX) and Santa Cruz Biotechnology (Dallas, TX) respectively; the antibody against talin1 was obtained from EMD Millipore (Billerica, MA).

### Western blot analysis

Cell lysates from prostate cells were subjected to Western blotting as previously described [58]. Subcellular fractionation was performed using NE-PER nuclear-cytoplasmic fraction kit (Thermo Scientific, Rockford, IL). Protein samples were analyzed by SDS-PAGE and transferred to Hybond-C membranes (Amersham Pharmacia Biotech, Inc; Piscataway, NJ). Membranes were incubated with the respective primary antibody (4°C), and exposed to species-specific peroxidase-labeled secondary antibodies. Signal detection was achieved and visualized using a UVP Imaging System. Protein bands were normalized to GAPDH expression.

### Cell viability assay

Cell viability was evaluated using the Thiazolyl Blue Tetrazolium bromide (MTT, Thermo Fisher Scientific, Waltham, MA) assay as previously described [8]. Briefly cells were seeded into 24-well plates and after grown to 60% to 75% confluence, were treated with vehicle or DZ-50 at indicated doses (DMSO, Sigma-Aldrich, St. Louis, MO). Absorbance was measured at 570nm and 690nm using μQuant Spectrophotometer (Biotech Instruments Inc., Winooski, VT).

### Quantitative RT-PCR analysis

RNA was extracted with the TRIzol reagent (Life Technologies, Waltham, MA), and RNA samples (1μg) were subjected to reverse transcription using the Reverse Transcription System (Promega, Madison, WI) [8]. TaqMan real-time RT-PCR (Life Technologies, Waltham, MA) analysis of the cDNA samples was conducted in an ABI7700 Sequence Detection System (Life Technologies, Waltham, MA) using the specific primers: E-cadherin (CDH1; Hs01023894_m1), N-cadherin (CDH2; Hs00983-056_m1), Snail (SNAI1; Hs00195591_m1), Slug (SNAI2; Hs00161904_m1), Zeb1 (ZEB1; Hs00232783_m1), vimentin (VIM; Hs00185584_m1), IGFBP3 (IGFBP3; Hs00365742_g1), and 18S rRNA (4319413E; Applied Biosystems, Life Technologies, Waltham, MA). Data represent average values from three independent experiments; numerical data normalized to 18s rRNA.

### RNA silencing

The siRNA knocking down IGFBP3 was obtained from Dharmacon (Lafayette, CO) with negative control sequence included. LNCaPTβRII cells were transfected either with a human IGFBP3 SMAT pool siRNA (GCUACAAAGUUGACUACGA; GAAAUG-CUAGUGAGUCGGA; GCACAGAUACCCAGAACUU; GAAUAUGGUCCCUGCCGUA; 50 ng/ml) or with an NC pool siRNA (UGGUUUACAUGUCGACUAA; UGGUUUACAUGUUGUGUGA; UGGUUUACAUGU-UUUCUGA; UGGUUUACAUGUUUUCCUA; 50 ng/ml;) in a solution containing DharmaFECT 3 Transfection Reagent (1µl/ml in antibiotic-free RPMI medium), at 37°C for 48hrs. IGFBP3 silencing at the mRNA and protein level was assessed by RT-PCR and Western blot analysis respectively. Transfected cells were treated with DZ-50 (4µM) for 3hrs and RT-PCR assays were performed as above.

### Immunofluorescent confocal microscopy

Cells were plated (1×10^5^) on cover glasses in 6-well plates as previously described [8]. Cells were exposed to medium (RPMI1640 with 10% FBS) in the presence of DZ-50 (4 µM), TGF-β1 (5ng/ml), or in combination of the two agents. Following treatment, cells were fixed in 4% paraformaldehyde and permeabilized with 0.1% Triton X-100 in sterile PBS. Fixed cells were incubated overnight with primary antibody specific for IGFBP3 and E-cadherin (Santa Cruz Biotechnology, Dallas, TX; BD Biosciences, San Jose, CA) at 4°C and the appropriate Alexa-Fluor (Life Technologies, Waltham, MA) fluorescent secondary antibody (1hr, room temperature). Slides were mounted using Vectashield mounting medium with DAPI (Vector Laboratories, Inc. Burlingame, CA) and were visualized using a FV1000 Confocal Microscope (Markey Cancer Center Core, University of Kentucky, Lexington, KY).

### Cell migration assay

CellTracker Green CMFDA dye (5 μmol/L; Invitrogen, Waltham, MA) dissolved in DMSO were added to flask culturing CAF (45mins, 37°C) as previously described [58]. CellTracker Orange CMTML (Invitrogen, Waltham, MA) in 1640 RPMI was added to prostate cancer cells, DU-145, DU-145talin1 and LNCaPTβRII, (45 mins, 37°C). Labeled cell suspensions of CAFs were placed into the 24-well plate included in the Biocat Matrigel Transwell Chamber (Corning, Bedford, MA). Labeled prostate cancer epithelial cells were seeded respectively in the transwell inserts placed (in 24-well plates) in the absence or presence of DZ-50, and/or TGF-β1 ligand (5 ng/mL, R&D System, Minneapolis, MN), and TGF-β1 neutralizing antibody (NA; R&D System, Minneapolis, MN). Invading prostate cancer cells were visualized using an epifluorescence Nikon Eclipse E600 microscope (Nikon, Melville, New York).

### Statistical analysis

The Student *t*-test, one-way, or two-way ANOVA were performed using GraphPad Prism 6 software to determine the statistical significance of difference between means/treatments. All numerical data are presented as mean± SEM. Statistical significance was set at *p* < 0.05.

## SUPPLEMENTARY MATERIALS FIGURES 



## Abbreviations

ADT: androgen deprivation therapy
CAFs: cancer-associated fibroblasts
CRPC: castration-resistant prostate cancer
EMT: epithelial-mesenchymal transition
TGF-β1: transforming growth factor-β1
IGF: insulin-like growth factor
IGFBP3: insulin growth factor binding protein-3
LNCaPTβRII: LNCaP cells overexpressing TGF-β receptor II
MET: mesenchymal-epithelial transition
ZEB1: E-box-binding protein1
