# Supplementary material for: Reversion of epithelial-mesenchymal transition by a novel agent DZ-50 via IGF binding protein-3 in prostate cancer cells
Source: Oncotarget. 2017 Jul 28;8(45):78507–19. doi: 10.18632/oncotarget.19659 (PMC5667978; doi:10.18632/oncotarget.19659)
Supplement: Supplementary file 1 [file oncotarget-08-78507-s001.pdf]

Reversion of epithelial-mesenchymal transition by a novel agent DZ-50 via IGF binding protein-3 in prostate cancer cells

SUPPLEMENTARY MATERIALS

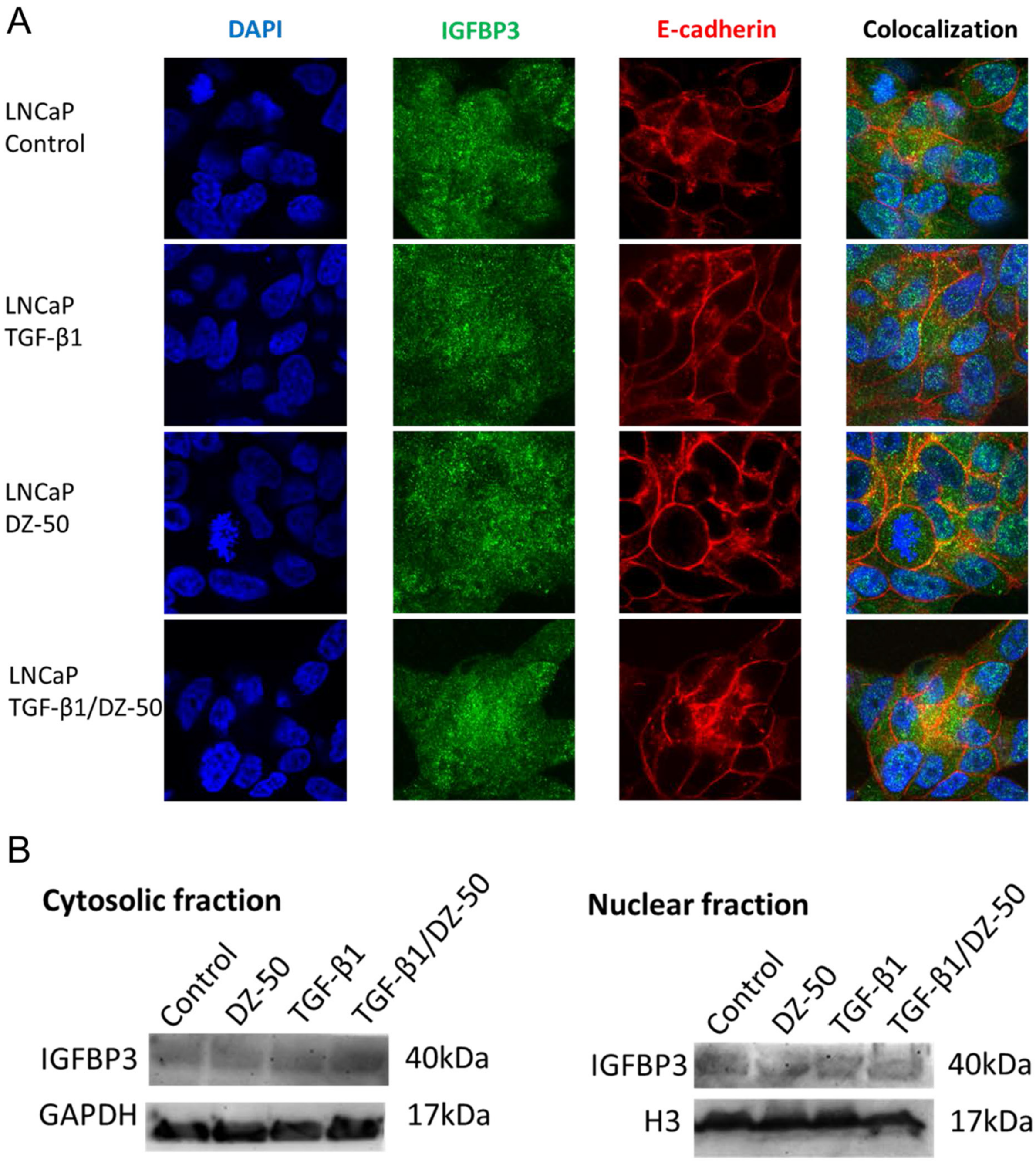

Supplemental Figure 1: The effect of TGF-β1 (5ng/ml), DZ-50 (4μM), and the two reagents in combination after 24 hrs on IGFBP3 and E-cadherin localization using confocal microscopy **(Panel A)** and protein expression using Western Blot analysis **(Panel B)** in LNCaP cells.

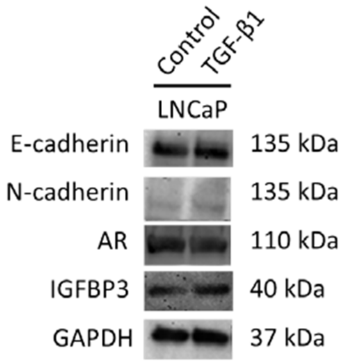

Supplementary Figure 2: The effect of TGF-β1 (5ng/ml, 48hrs) on E-cadherin, N-cadherin, AR, IGFBP3 protein expression levels in LNCaP cells.

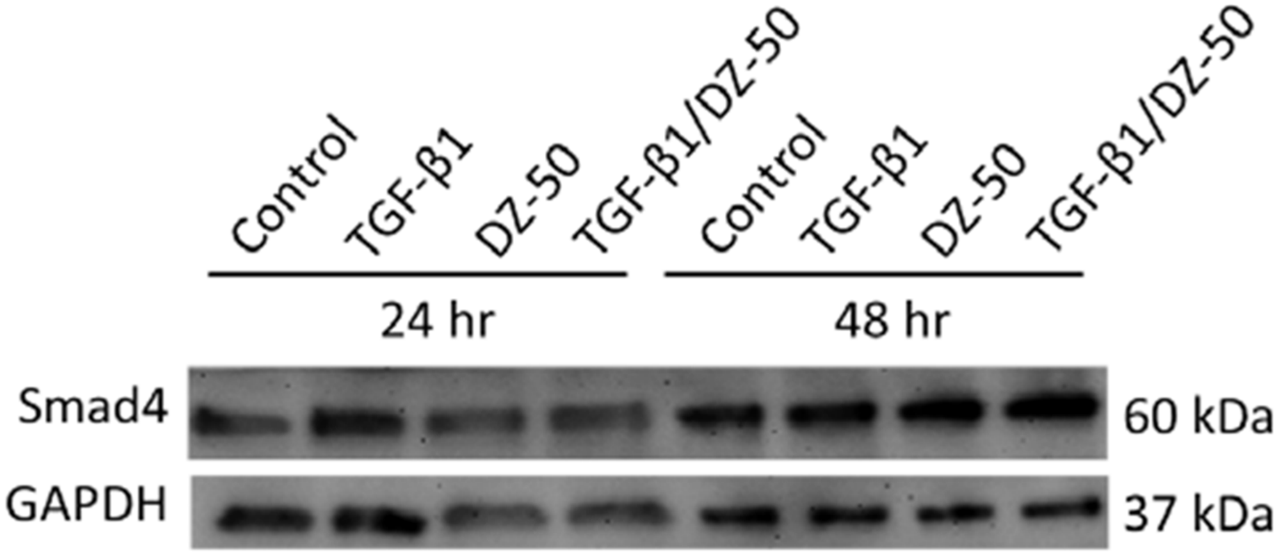

Supplementary Figure 3: Western blot analysis of Smad4 expression in LNCaPTβRII cells in response to DZ-50 (4μM, 48hrs), in the presence or absence of TGF-β1 (5ng/ml, 48hrs).

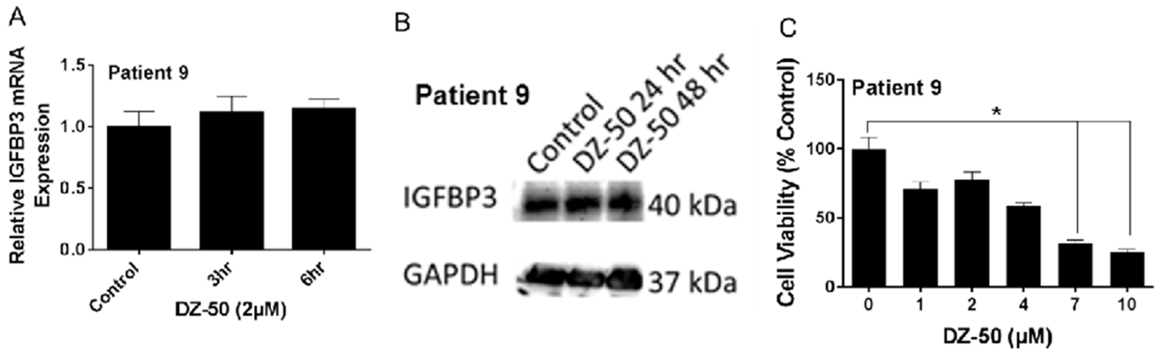

Supplementary Figure 4: The effect of DZ-50 on IGFBP3 mRNA and protein expression in prostate CAFs (derived from patient 9). **Panel A**, shows IGFBP3 mRNA expression after 3 and 6 hrs DZ-50 treatment (2μM). n=6. **Panel B**, shows IGFBP3 protein expression after 24 and 48hrs DZ-50 treatment. **Panel C**, shows the effect of DZ-50 treatment on cell viability. n=3. * $p<0.05$.

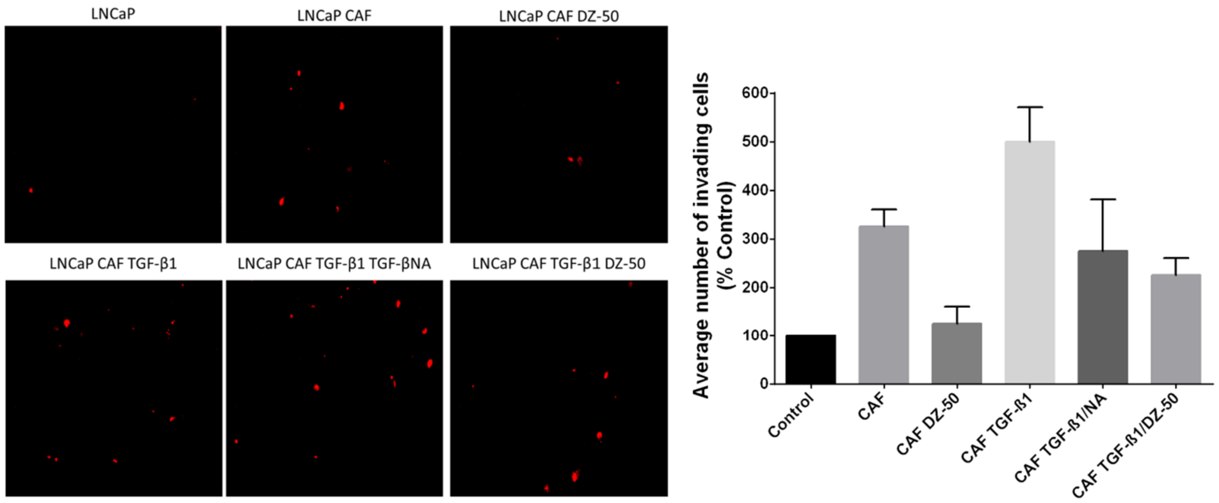

Supplementary Figure 5: Impact of DZ-50 treatment on CAF-mediated migration of LNCaP cells.

Supplementary Table 1: CAF and tumor cell lysates were derived from 6 patients in the following table.

Patients	Age	Race	Family history	Gleason score	% Prost involv	PSA at DX
#1	67	AA	No	3+4	10	3.2
#2	55	AA	No	3+4	10	5.7
#4	56	AA	Yes (Uncle)	3+4	15	1.48
#5	63	AA	Yes (father &brother	4+3	45	35
#9	60	AA		4+5	75	18.5
#10	51	AA	no	3+4	20	
